# A Practical Overview of the Stool DNA Test for Colorectal Cancer Screening

**DOI:** 10.14309/ctg.0000000000000464

**Published:** 2022-04-05

**Authors:** Sanya Anand, Peter S. Liang

**Affiliations:** 1 SUNY Downstate Health Sciences University, Brooklyn, New York, USA;; 2 NYU Langone Health, VA New York Harbor Health Care System, New York, New York, USA.

## Abstract

The multitarget stool DNA test with fecal immunochemical test (sDNA-FIT) is recommended by all major US guidelines as an option for colorectal cancer screening. It is approved by the Food and Drug Administration for use in average-risk individuals aged 45 years and older. The sDNA-FIT tests for 11 biomarkers, including point mutations in *KRAS*, aberrant methylation in *NDRG4* and *BMP3*, and human hemoglobin. Patients collect a stool sample at home, send it to the manufacturer's laboratory within 1 day, and the result is reported in approximately 2 weeks. Compared with FIT, sDNA-FIT has higher sensitivity but lower specificity for colorectal cancer, which translates to a higher false-positive rate. A unique feature of sDNA-FIT is the manufacturer's comprehensive patient navigation system, which operates 24 hours a day and provides active outreach for patient education and reminders in the first month after a test is ordered. Retesting is recommended every 1–3 years, although the optimal testing interval has not yet been determined empirically. The cost of sDNA-FIT is $681 without insurance, but Medicare and most private insurers cover it with no copay or deductible.

## INTRODUCTION

Colorectal cancer (CRC) is the third most common cancer and the third leading cause of cancer deaths in both women and men in the United States, with an estimated 149,500 new cases and 52,980 deaths in 2021 (1). Overall CRC incidence and mortality have declined in the past few decades, which are partially attributed to the effectiveness of screening (2). However, CRC incidence among individuals younger than 50 years has been increasing (3), which led the US Preventive Services Task Force (USPSTF) to lower the recommended starting age for screening in average-risk persons to 45 years in 2021 (4).

Up-to-date screening uptake among adults aged 50–75 years rose to 68.8% in 2018 (5) but still fell well short of the 80% goal set by the National Colorectal Cancer Roundtable. In addition, screening uptake was substantially lower in younger individuals and minority racial/ethnic groups such as Hispanics, American Indians/Alaska Natives, and Asians/Pacific Islanders. Screening methods recommended by the USPSTF can be categorized as either direct visualization or noninvasive stool tests, and the latter includes guaiac-based fecal occult blood testing (gFOBT), fecal immunochemical testing (FIT), and the multitarget stool DNA test with an FIT component (mt-sDNA or sDNA-FIT). In this article, we provide a practical overview of the sDNA-FIT for the practicing gastroenterologist.

## TEST OVERVIEW

The biological basis of stool DNA tests rests on the fact that colorectal neoplasms continuously shed DNA, which can be detected in the stool after amplification (6). The only sDNA-FIT approved by the US Food and Drug Administration (Cologuard, Exact Sciences Corporation) evaluates 11 biomarkers—7 point mutations in the *KRAS* gene, 2 methylation markers of the *NDRG4* and *BMP3* genes, β-actin as a control for human DNA quantity, and human hemoglobin using an FIT (7).

**Figure 1.**
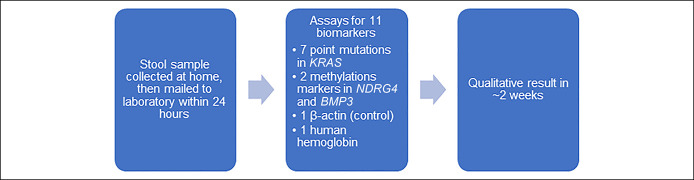
Timeline and process for sDNA-FIT. sDNA-FIT, stool DNA test with fecal immunochemical test.

The sDNA-FIT is ordered by a healthcare provider—or a telemedicine provider available through the product website—and sent directly to the patient by the manufacturer. As with the FIT, patients do not need to adjust their diet or medications to use the sDNA-FIT. Patients collect a stool sample in a container, use a probe to obtain a second smaller sample for the FIT, pour a preservative into the container, and mail both specimens back to the manufacturer's laboratory within 24 hours (Figure 1). The manufacturer offers a patient and provider navigation system with interpreter services, which operates 24 hours a day and features active outreach for patient education and reminders in the first 30 days after a test is ordered (8,9).

Results of the DNA biomarker tests are incorporated into a logistic regression algorithm with a predetermined cutoff for positive values. The overall sDNA-FIT result is reported as positive if either the DNA biomarkers or the FIT result is positive, and test results are delivered to the prescribing provider usually within 2 weeks. Patients with a positive sDNA-FIT result should undergo a follow-up colonoscopy, whereas those with a negative test result should repeat CRC screening every 1–3 years (4,10).

The US Food and Drug Administration (FDA) approved sDNA-FIT for CRC screening in 2014 and expanded the approved age range to individuals 45 years and older in 2019. It is currently available only in the United States. The sDNA-FIT was included as a screening option in the 2016 USPSTF and 2018 American Cancer Society (ACS) guidelines (10,11).

## INDICATIONS

The sDNA-FIT is indicated for asymptomatic adults aged 45 years and older who are at an average risk for CRC. Individuals with symptoms and asymptomatic persons at a higher risk—including those with a personal history of colorectal neoplasia, hereditary cancer syndromes, inflammatory bowel disease, or a first-degree relative diagnosed with advanced colorectal neoplasia before age 60 years—are recommended to undergo colonoscopy.

## PERFORMANCE CHARACTERISTICS

A pivotal cross-sectional study of 9,989 average-risk individuals aged 50–84 years at 90 sites across the United States and Canada—who underwent both sDNA-FIT and FIT, followed by colonoscopy—provided the definitive performance characteristics of the sDNA-FIT (Table 1) (12). The study identified 65 participants with CRC and an additional 757 participants with advanced precancerous lesions, including advanced adenomas and sessile serrated lesions measuring 10 mm or greater. The sDNA-FIT positivity was 16%. Sensitivity for CRC and advanced precancerous lesions was 92.3% and 42.4% for sDNA-FIT, compared with 73.8% and 23.8% for FIT, respectively. For those without CRC or advanced precancerous lesions, specificity was 86.6% for sDNA-FIT and 94.9% for FIT. Given the 0.7% prevalence of CRC in the study population, the positive predictive value (PPV) or likelihood that an individual with a positive test was diagnosed with CRC was 3.7% for sDNA-FIT and 6.9% for FIT. For advanced colorectal neoplasia, which include CRC and advanced precancerous lesions, the prevalence was 8.2%, and the PPV was 23.6% for sDNA-FIT and 32.6% for FIT. The negative predictive value (NPV) or likelihood a person with a negative test result did not have CRC was 99.9% for sDNA-FIT and 99.8% for FIT. The NPV for advanced colorectal neoplasia was 94.7% for sDNA-FIT and 93.6% for FIT. The number of persons needed to undergo screening to detect one case of CRC, which is a measure of both disease prevalence and test sensitivity, was 166 for sDNA-FIT and 208 for FIT.

**Table 1.** Test characteristics for sDNA-FIT and FIT

Test characteristic	sDNA-FIT	FIT
Sensitivity (CRC)	92.3%	73.8%
Sensitivity (advanced precancerous lesions)^a^	42.4%	23.8%
Specificity (CRC and advanced precancerous lesions)	86.6%	94.9%
Specificity (CRC and all precancerous lesions)	89.8%	96.4%
Positive predictive value (CRC)	3.7%	6.9%
Negative predictive value (CRC)	99.9%	99.8%
False-positive rate (CRC and advanced precancerous lesions)	13.4%	5.1%
False-negative rate (CRC)	7.7%	26.2%
Test positivity	16.1%	7.0%
Number of persons needed to screen to detect 1 CRC	166	208
Testing interval	3 yr	1 yr

CRC, colorectal cancer; sDNA-FIT, stool DNA test with fecal immunochemical test.

a Advanced precancerous lesions: advanced adenoma and sessile serrated lesions 10 mm or larger.

In a smaller study of 661 Alaska Native adults aged 40–85 years, sensitivity for advanced colorectal neoplasia in those undergoing screening was also higher for sDNA-FIT compared with that for FIT (50% vs 31%, *P* = .01) (13). Conversely, specificity in individuals without advanced colorectal neoplasia was lower for sDNA-FIT than for FIT (91% vs 94%, *P* = .02). Given the younger (median age 55 years) and ethnically distinct population of the Alaska study, the similarity of findings with the larger pivotal study provided further evidence of generalizability across broad age and racial/ethnic demographic groups.

In 2021, a cross-sectional study of 816 individuals aged 45–49 years demonstrated the performance characteristics of sDNA-FIT for the youngest population eligible for screening. Specificity for advanced colorectal neoplasia, the primary outcome, was 95.2% (14). No cases of CRC and 49 cases of advanced precancerous lesions were detected, and sensitivity for advanced precancerous lesions was 32.7%.

## LIMITATIONS

Similar to other stool-based screening tests, patients need to collect their own samples at home. Compared with FIT, sDNA-FIT has a more complex collection and diagnostic process that requires a larger stool specimen, a liquid preservative, and 2 additional molecular assays. These differences likely explain the 6-fold higher technical failure rate for sDNA-FIT compared with that for FIT (213 vs 34 cases) in the pivotal study (12). It is unclear how frequently invalid sDNA-FIT results occur in clinical practice and whether the need for repeat testing affects overall uptake. However, in a study of 368,494 Medicare beneficiaries who were prescribed the sDNA-FIT, 71% completed the test within 1 year, and the median time to test completion was 27 days (9).

As with all noninvasive screening modalities, a positive sDNA-FIT result requires a follow-up colonoscopy to complete the testing strategy. Although the manufacturer built a patient navigation system to ensure the sDNA-FIT is completed, this system does not facilitate the colonoscopy. In a large integrated system in which 1,242 individuals had positive sDNA-FIT results, 69% completed colonoscopy within 6 months and 73% completed colonoscopy within 1 year (15). These findings underscore the importance and challenge of completing a timely colonoscopy after a positive sDNA-FIT result.

Because sDNA-FIT assesses 10 biomarkers in addition to hemoglobin, providers may have greater concern that a positive test, followed by a negative colonoscopy, still requires an additional follow-up. However, in a cohort of 205 individuals who had a positive sDNA-FIT result, followed by a negative colonoscopy without advanced colorectal neoplasia in the pivotal study, incidence of aerodigestive cancer was similar compared with the general population after a median 5.3 years of follow-up (16). These results suggest that individuals with false-positive sDNA-FIT results do not require further testing.

## INCORPORATION INTO CLINICAL CARE

As with other stool-based screening tests, primary care providers order the most sDNA-FIT. In the large study of Medicare beneficiaries, 88% of sDNA-FIT were ordered by primary care providers, whereas gastroenterologists led the other specialties with 6% of orders (9). Notably, test completion rate was higher for those ordered by gastroenterologists than primary care providers (78% vs 71%, respectively), suggesting that patients may especially value screening recommendations from gastroenterologists. The product website provides comprehensive information on how to order sDNA-FIT, which can be performed through fax, the electronic medical record, or the online EpicCare Link tool (17). Test order status reports and test results are also available by fax or electronically.

The optimal screening interval for sDNA-FIT has not yet been established by data. Although the FDA approved sDNA-FIT based on a 3-year testing interval, it stipulated that the manufacturer must conduct a postapproval study to empirically assess this interval. Results of this prospective longitudinal study (NCT02419716) are pending and will show the PPV and NPV of sDNA-FIT in the second round of screening. The ACS currently recommends repeating the test every 3 years (10), although the modeling study used to inform its guidelines found sDNA-FIT was not a model-recommended strategy (18). USPSTF recommends a 1 to 3-year testing interval (4), even though the modeling it commissioned found only 1- to 2-year testing intervals for sDNA-FIT were efficient or near-efficient compared with FIT strategies (19). However, because Medicare and many private insurers currently cover sDNA-FIT every 3 years, this will remain the default testing interval unless new data emerge.

## ALTERNATIVES

In both the 2018 ACS and 2021 USPSTF CRC screening guidelines, sDNA-FIT is presented as one of 6 equally recommended tests, along with FIT, gFOBT, colonoscopy, sigmoidoscopy, and CT colonography (4,10). While any of the other 5 tests can be considered alternatives to sDNA-FIT for CRC screening, in practice, FIT and, to a lesser extent, gFOBT are regarded as its main competitors because they are all stool-based tests.

The advantages of sDNA-FIT compared with those of FIT include a 19% higher sensitivity for both CRC and advanced precancerous lesions (12), a built-in patient navigation system to increase testing adherence, and a longer testing interval as currently used in practice (3 years for sDNA-FIT vs 1 year for FIT).

On the contrary, the disadvantages of sDNA-FIT include an 8% lower specificity and a lower PPV for advanced colorectal neoplasia compared with those of FIT. This translates to a higher false-positive rate, meaning a smaller proportion of diagnostic colonoscopies will find the most clinically significant lesions. The greater technical complexity and the higher cost of sDNA-FIT, as detailed in the next section, precludes its use for mass outreach campaigns such as mailed FIT or in uninsured populations. Finally, unlike gFOBT (20,21), the relatively new sDNA-FIT lacks clinical evidence that it reduces CRC mortality or incidence.

There are data to suggest that patients prefer sDNA-FIT over both FIT and colonoscopy. In a nationally representative survey study of 1,062 average-risk individuals aged 45–75 years, 61% of respondents had heard of sDNA-FIT and 14% had used the test, compared with 67% and 29% for FIT or gFOBT and 91% and 57% for colonoscopy, respectively (22). When asked about their preferences between pairs of tests, most of them chose sDNA-FIT (65%) and FIT/gFOBT (61%) over colonoscopy, and 67% chose sDNA-FIT over FIT/gFOBT.

## BILLING, COST, AND COST-EFFECTIVENESS

The sDNA-FIT is billed under CPT code 81528. All tests are processed at Exact Sciences Laboratories (NPI 1629407069). For individuals without insurance, the out-of-pocket cost for sDNA-FIT is $681. The test is covered with no copay or deductible for Medicare patients aged 50–85 years. Medicaid coverage varies by state. Most private insurers cover sDNA-FIT with no copay or deductible for individuals aged 50–75 years. According to the manufacturer, more than 94% of individuals overall and more than 80% of those aged 45–49 years who are ordered the test incur no out-of-pocket cost (23).

However, it is essential to point out that individuals who need a colonoscopy to follow-up a positive sDNA-FIT result—or any other noncolonoscopy screening test—may face a substantial bill. This is because while Medicare and most private insurers cover a number of CRC screening tests with no cost-sharing, a colonoscopy to follow-up another positive test has been considered a diagnostic procedure and may incur out-of-pocket costs. New federal guidance issued in January 2022 mandated that private insurers can no longer impose cost-sharing for a follow-up colonoscopy after a positive noncolonoscopy test starting May 31, 2022. However, this guidance does not apply to Medicare.

Although organizations such as the ACS and the USPSTF do not consider cost-effectiveness in their guidelines, a recent cost-effectiveness study was conducted using one of the microsimulation models used by both organizations (24). The study assessed 4 alternatives tests to colonoscopy and FIT—sDNA-FIT, CT colonography, colon capsule endoscopy, methylated septin 9 blood test—and concluded that sDNA-FIT every 3 years was not cost-effective. Annual sDNA-FIT had an incremental cost-effectiveness ratio per quality-adjusted life-year gained of $214,974, which exceeded the commonly used willingness-to-pay threshold of $100,000. Screening with sDNA-FIT every 1 or 3 years was more expensive than either annual FIT or colonoscopy every 10 years, and annual sDNA-FIT was the most expensive of all 10 strategies considered. Therefore, whereas sDNA-FIT may not result in any out-of-pocket expenses for most individuals with insurance, from a societal perspective, this screening strategy would add substantial cost to the healthcare system.

## CONCLUSION

Since obtaining FDA approval in 2014, sDNA-FIT has become one of the 6 CRC screening tests recommended by all major US guidelines. It is a noninvasive, stool-based test with higher sensitivity but lower specificity for CRC than FIT. With a built-in patient navigation system and a longer screening interval compared with FIT, sDNA-FIT addresses key barriers to adherence in ways that will improve uptake. However, the high cost of sDNA-FIT may limit its use, especially for uninsured and underinsured populations.

## CONFLICTS OF INTEREST

**Guarantor of the article:** Peter S. Liang, MD, MPH.

**Specific author contributions:** S.A. and P.S.L.: planned the study, performed the literature review, and drafted the manuscript. Both authors approved the final draft to be submitted.

**Financial support:** No external grant funds were used in this study.

**Potential competing interests:** P.S.L. has received research support from Epigenomics and Freenome and serves as an advisory board member to Guardant Health. S.A. has no conflicts to declare.
